# *Schistosoma* *japonicum* Eggs Induce a Proinflammatory, Anti-Fibrogenic Phenotype in Hepatic Stellate Cells

**DOI:** 10.1371/journal.pone.0068479

**Published:** 2013-06-19

**Authors:** Barrie J. Anthony, Kylie R. James, Geoffrey N. Gobert, Grant A. Ramm, Donald P. McManus

**Affiliations:** 1 Molecular Parasitology Laboratory, Queensland Institute of Medical Research, Brisbane, Australia; 2 The Hepatic Fibrosis Group, Queensland Institute of Medical Research, Brisbane, Australia; 3 Faculty of Health Sciences, The University of Queensland, Brisbane, Australia; Northwestern University Feinberg School of Medicine, United States of America

## Abstract

Hepatic fibrosis induced by egg deposition is the most serious pathology associated with chronic schistosomiasis, in which the hepatic stellate cell (HSC) plays a central role. While the effect of *Schistosoma mansoni* eggs on the fibrogenic phenotype of HSCs has been investigated, studies determining the effect of eggs of *S* *. japonicum* on HSCs are lacking. Disease caused by *S* *. japonicum* is much more severe than that resulting from *S. mansoni* infection so it is important to compare the pathologies caused by these two parasites, to determine whether this phenotype is due to the species interacting differently with the mammalian host. Accordingly, we investigated the effect of *S* *. japonicum* eggs on the human HSC cell line, LX-2, with and without TGF-β (Transforming Growth Factor beta) co-treatment, so as to determine the impact on genes associated with fibrogenesis, inflammation and matrix re-organisation. Activation status of HSCs was assessed by αSMA (Alpha Smooth Muscle Actin) immunofluorescence, accumulation of Oil Red O-stained lipid droplets and the relative expression of selected genes associated with activation. The fibrogenic phenotype of HSCs was inhibited by the presence of eggs both with or without TGF-β treatment, as evidenced by a lack of αSMA staining and reduced gene expression of αSMA and Col1A1 (Collagen 1A1). Unlike *S. mansoni*-treated cells, however, expression of the quiescent HSC marker PPAR-γ (Peroxisome Proliferator-Activated Receptor gamma) was not increased, nor was there accumulation of lipid droplets. In contrast, *S* *. japonicum* eggs induced the mRNA expression of MMP-9 (Matrix Metalloproteinase 9), CCL2 (Chemokine (C-C motif) Ligand 2) and IL-6 (Interleukin 6) in HSCs indicating that rather than inducing complete HSC quiescence, the eggs induced a proinflammatory phenotype. These results suggest HSCs in close proximity to *S* *. japonicum* eggs in the liver may play a role in the proinflammatory regulation of hepatic granuloma formation.

## Introduction

Schistosomiasis is the most important of the human helminthiases, estimated to infect 200 million people resulting in a loss of millions of disability-adjusted life-years (DALYs) per annum [1–3]. Infection with *Schistosoma* *japonicum* , is associated with chronic liver and intestinal fibrosis, with approximately one million people infected and another 50 million at risk in China [4]. The pathology arises predominantly from parasite eggs, many of which become trapped in the host liver, and is associated with both acute and chronic forms of schistosomiasis [4]. Chronic disease is hepatosplenic associated, producing a granulomatous reaction to eggs trapped in the host’s liver [5]. The resulting liver fibrosis eventually contributes to blood flow occlusion through the liver, leading to portal hypertension, splenomegaly, portacaval shunting, ascites, gastrointestinal varices, gastrointestinal bleeding and death [5].

The mechanisms driving hepatic fibrosis in schistosomiasis are becoming better understood. The fibrosis is driven by IL-13 production, with IL-13 knockout mice developing less fibrosis [6]. Similarly fibrosis is reduced by increased expression of the IL-13 decoy receptor [6]. IL-13 has been demonstrated to stimulate collagen production in a human hepatic stellate cell (HSC) line [7], as well as in primary rat HSCs [8]. The HSC is one of the main cells contributing to fibrosis within the liver and has a contributory role in collagen production in murine and human *S* *. japonicum* infections [9] and human *S. mansoni* infection [10]. HSCs are located within the space of Dissé in the sinusoid where they are responsible for vitamin A storage and maintenance of a low density matrix within this space [11]. In response to insult or injury, HSCs undergo a process of transdifferentiation, becoming fibrogenic myofibroblasts responsible for collagen production and accumulation of a scar-like matrix [12]. This process is well understood *in vitro* with primary HSCs undergoing spontaneous activation in normal cell culture conditions, which has allowed the identification of markers of activation status. Quiescent cells are associated with lipid droplet retention and increased gene expression of peroxisome proliferator-activated receptor gamma (PPAR-γ) [13,14], while activated cells express fibrogenesis-associated genes, have little lipid droplet retention, but do display increased stress fibres, particularly α smooth muscle actin (αSMA) [15]. As human primary cells are difficult to isolate, cell lines have been developed to enable the study of human HSC interactions [16]. The LX-2 cell line has been demonstrated to retain many features of primary HSC cells [16]. One of the main known activators of these cells is TGF-β (Transforming Growth Factor beta) and its expression has been linked to a number of diseases associated with liver fibrosis [17–19]. The response to TGF-β is well documented and is used as an *in vitro* model for HSC activation [20,21] and previously on LX-2 cells [16].

It has been previously demonstrated that eggs of *S. mansoni* can reverse HSC transdifferentiation, promoting the quiescent phenotype, supporting the theory that fibrosis is host-driven [15]. In that particular study, schistosome eggs were co-cultured with LX-2 cells and biomarkers of transdifferentiation measured. *S. mansoni* eggs reduced the expression of αSMA and collagen (Col1A1), but promoted PPARγ expression resulting in a more quiescent morphology, as characterised by the lack of stress fibre staining and an increased accumulation of lipid droplet storage, when compared with cells cultured without eggs [15]. Our present study investigated the effects of *S* *. japonicum* eggs on the transdifferentiation status of LX-2 cells and standard biomarkers of HSC activation. *S* *. japonicum* causes much more severe disease than *S. mansoni*, which may be due to differences in the interaction of the eggs of the two schistosome species on HSCs thereby influencing disease outcome. We found that while eggs of *S* *. japonicum* decreased fibrogenesis in the cells, observed by reduced mRNA expression of αSMA and Col1a1 accompanied by a loss of αSMA stress fibres, there was no associated increase in expression of PPARγ and the cells failed to accumulate lipid droplets. While causing an anti-fibrogenic phenotype in HSCs, *S* *. japonicum* eggs induced a significant increase in the gene expression of the proinflammatory mediators MMP9, CCL2 (chemokine (C-C motif) ligand 2) and IL-6 (Interleukin 6), suggesting a potential role in the regulation of granuloma development via inflammatory cell recruitment and matrix remodelling.

## Materials and Methods

### Animal Ethics

The conduct and procedures involving animal experiments were approved by the Animals Ethics Committee of the Queensland Institute of Medical Research (project no. P288). This study was performed in accordance with the recommendations of the Australian code of practice for the care and use of animals for scientific purposes, 2004.

### Isolation of *S* *. japonicum* eggs

Swiss outbred mice (aged 8-12 weeks) were percutaneously challenged with 60 *S* *. japonicum* cercariae (Mainland Chinese strain, Anhui population) and, at 6 weeks post-infection, the livers were harvested for eggs. All egg extraction procedures were performed under aseptic conditions as previously described [15]. Approximately five infected mouse livers were pooled, finely cut up and digested in approximately 5mL of digestion buffer (8.95g NA _2_HPO_4_, 0.45g KH_2_PO_4_ in 1L milli-Q H_2_0) and 2g of trypsin (Sigma, Australia) for 3h at 37^°^C. The homogenate containing the eggs was passed through a series of sieves (250-160μm), the eggs were collected by sedimentation and then purified by washing six times in sterile phosphate-buffered saline. The purified eggs were then counted and re-suspended in DMEM (Invitrogen, Australia) cell culture medium. The eggs were stored in DMEM with 10% (v/v) foetal bovine serum (FBS, Lonza, Australia) overnight before use the next day. The egg preparation was tested with the Limulus Amebocyte Lysate assay (Lonza, Australia) and confirmed free of LPS. HSC were then co-cultured for up to 3 days in the presence of the viable eggs at a concentration of 1000 eggs/ml. Egg viability was tested by a hatching assay.

### LX2 Cell Culture

The LX-2 cell line is a well established human HSC cell line [16]. Cultures were maintained in DMEM containing 2% (v/v) FBS plus antibiotics (complete medium) at 37^o^C and with 5% CO_2_. Medium was changed after 48 hours. Cells of the human HSC cell line LX-2 were seeded in 24 well plates (BD falcon, Australia) at a density of 4.3x10^3^ cells per cm^2^. For co-culture experiments with *S* *. japonicum* eggs, cells were treated for up to 3 days with either complete medium alone or complete medium + eggs, either in direct contact with the cells or separated from the cells by use of an cell culture insert within a 24 well plate transwell plate system (Sigma, Australia). For experiments with recombinant TGF-β1, cells were treated with either complete medium + TGF-β1 (Cell Signalling Technologies, Australia) (5ng/ml), or with complete medium + *S* *. japonicum* eggs (1000 eggs/ml) + TGF-β1 (5ng/ml). In a preliminary study both *S. mansoni* and *S* *. japonicum* eggs were used in independent experiments. Results obtained with HSC co-cultured with *S. mansoni eggs*-were entirely consistent with those previously obtained [15]. Consequently, a side by side comparison was considered unnecessary but a general direct comparison was made of the results obtained with HSCs co-cultured with the eggs of *S* *. japonicum* and *S. mansoni* [15].

### Immunofluorescence and phase-contrast microscopy

Cells were fixed and stained for αSMA as previously described [15]. Cells for lipid droplet staining were fixed and stained with Oil Red O as previously described [15] A positive control was prepared by treating LX-2 cells with MDI(0.5mM isobutylmethylxanthine, 1μM dexamethasone and 1μM insulin) to induce lipid droplet formation as previously described [15]. Phase-contrast and immunofluorescence images were obtained with an Olympus CKX41 phase-contrast microscope and an Incell Analyser 2000 (GE Healthcare Life Sciences, city), respectively. Images of Oil-Red O stained lipid droplets were obtained using an Olympus CKX41 phase-contrast microscope.

### Real time PCR

LX-2 cells were seeded into 6 well plates (BD Falcon, Australia) with 10^5^ cells per well in complete medium. Once the cells reached ~70% confluence, the complete medium was replaced with DMEM containing 0.1% FBS and incubated overnight before treatment in the following morning. Treatments were then performed as described above. After 24 or 72h cells were lysed *in situ* using cell protect reagent (Qiagen, Australia) and stored at -80°C prior to RNA extraction. Total RNA was extracted, and genomic DNA removed by spin-column purification according to the supplier’s recommendations (RNeasy Mini Plus kit, Qiagen, Australia). RNA yield and purity were evaluated spectrophotometrically. Absorbance was measured at 260nm and 280nm and the purity was determined from the absorbance ratio (A260/A280). Total RNA (1μg) was used in a reverse transcription reaction and cDNA synthesis performed using a Quantitech reverse transcription kit according to the supplier’s instructions (Qiagen, Australia).

A one-step real time-PCR (qPCR) was conducted on the cDNA using the QuantiFast SYBR Green PCR Kit in a Rotogene 3000 to determine changes in cellular transcription of CTGF (Connective Tissue Growth Factor), α-SMA, PPARγ, Col1a1, Matrix Metalloproteinases (MMPs) 2 and 9, Interleukin 6 (IL-6), CCL2 and Tissue Inhibitor of Metalloproteinase 1 (TIMP1) relative to the house keeping genes YWHAZ and ATP5β [15] according to manufactures instructions. cDNA in 5 μl (100 ng in total) was added to 7.5 μl of primer master mix composed of 6.25 μl Rotor-Gene SYBR Green PCR Master Mix and 1.25 μl of the appropriate primer set. The qPCR run incorporated a hold for 5 min at 95^o^C followed by 40 cycles of 10 seconds at 95^o^C and 30 sec at 60^o^C and ended with a melt for 3 min at 95^o^C. Schistosome only RNA was used as a control and gave a negative result. Relative quantification was carried out via the delta delta cycle threshold (ΔΔCT) method [22] relative to the housekeeping genes built into REST2009 (Qiagen, Australia) software. All samples reactions were carried out using three biological replicates.

#### Statistical Analysis

Cell culture experiments were performed three times independently and representative images used. Gene experiments were performed twice independently. Statistical validation was performed within REST 2009 [22] using the Pair Wise Fixed Reallocation Randomisation Test and 10,000 randomisations were performed. Statistical significance was accepted when *p* ≤ 0.05.

## Results

### Effect of *S* *. japonicum* eggs on the fibrogenic phenotype of HSCs

**Figure 1:**
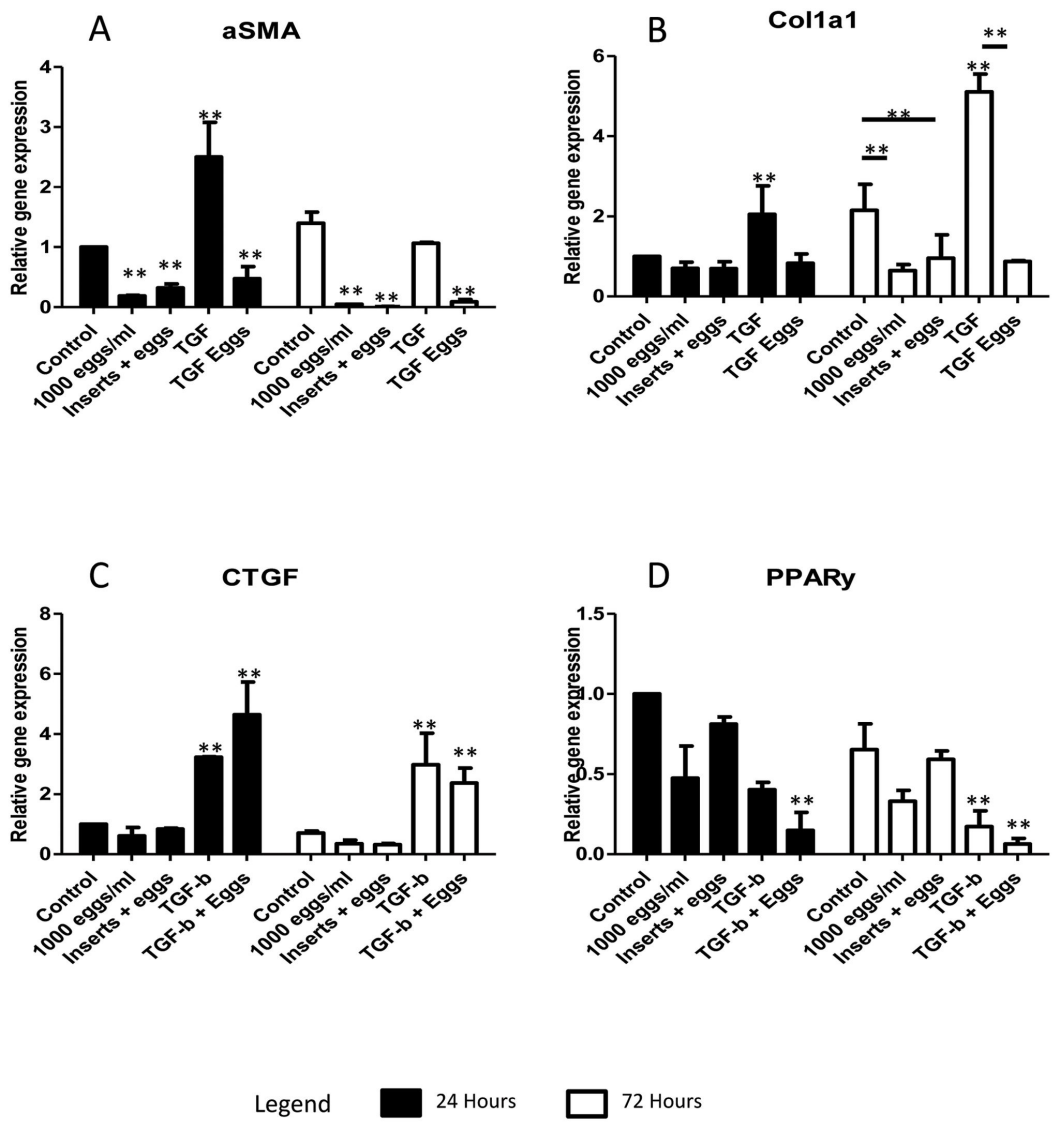
Effect of *S*. *japonicum* eggs on the activated HSC fibrogenic phenotype. A number of genes; A. αSMA, B. Col1A1, C. CTGF and D. PPARγ, were selected and their gene expression determined in LX-2 cells relative to the housekeeping genes YWHAZ and ATP5β. *S. japonicum* eggs, both in direct contact or separated by inserts from LX-2 cells, significantly inhibited expression of genes associated with fibrosis αSMA (A) and Col1a1 (B) compared with untreated cells. TGF-β treatment of cells resulted in significantly increased expression of αSMA (A), Col1a1 (B) and CTGF (C). The stimulatory effects of TGF-β on LX-2 cells were blocked by the presence of eggs, with the expression levels of these genes being similar to egg treatment alone, except in the case of CTGF (C) where increased expression levels where not inhibited by co-treatment*. S. japonicum* eggs failed to induce expression of PPARγ (n=3, SEM).

Treatment of LX-2 cells with *S* *. japonicum* eggs induced a significant (p value ≤ 0.05) decrease in the expression of αSMA at 24 h (5 fold) and both αSMA (20 fold) and Col1a1 (1.5 fold) at 72h after treatment (Figure 1A and B). This occurred whether eggs were in direct contact with LX-2 cells or whether eggs had no direct contact with the cells via inserts, indicating the effect observed was due to soluble products secreted from the eggs. TGF-β treatment of cells resulted in significant increases in the expression of αSMA (2.5 fold) at 24h and Col1A1 at both 24 h (2 fold) and 72h (5 fold) compared with the cells grown in normal cell culture conditions (Figure 1 A and B). However, co-treatment of cells with TGF-β and eggs resulted in down-regulation of these genes, similar to expression levels observed with cells treated with eggs alone. This demonstrates that the eggs can switch off the pro-fibrogenic response of LX-2 cells to TGF-β. CTGF expression was unchanged in cells cultured with eggs while PPARγ (a marker of HSC quiescence), was decreased, although not significantly (p value > 0.05), in cells cultured in the presence of eggs versus untreated controls (Figure 1 C and D). TGF-β significantly increased expression of CTGF mRNA (3 fold) in LX-2 cells by 72h, a response which not affected by egg treatment (Figure 1 C). There was a significant down-regulation in PPARγ mRNA expression in LX-2 cells by co-treatment with eggs and TGF-β. These results indicate that while the genes associated with the profibrogenic phenotype of HSCs were down-regulated by *S* *. japonicum* eggs, a switch to a fully quiescent phenotype did not occur as evidenced by the unchanged expression of PPARγ. In addition, the profibrogenic response to TGF-β was lost in LX-2 cells, possibly due to the induction of CTGF.

### Effect of *S* *. japonicum* eggs on matrix remodelling and inflammatory mediators in HSCs

**Figure 2:**
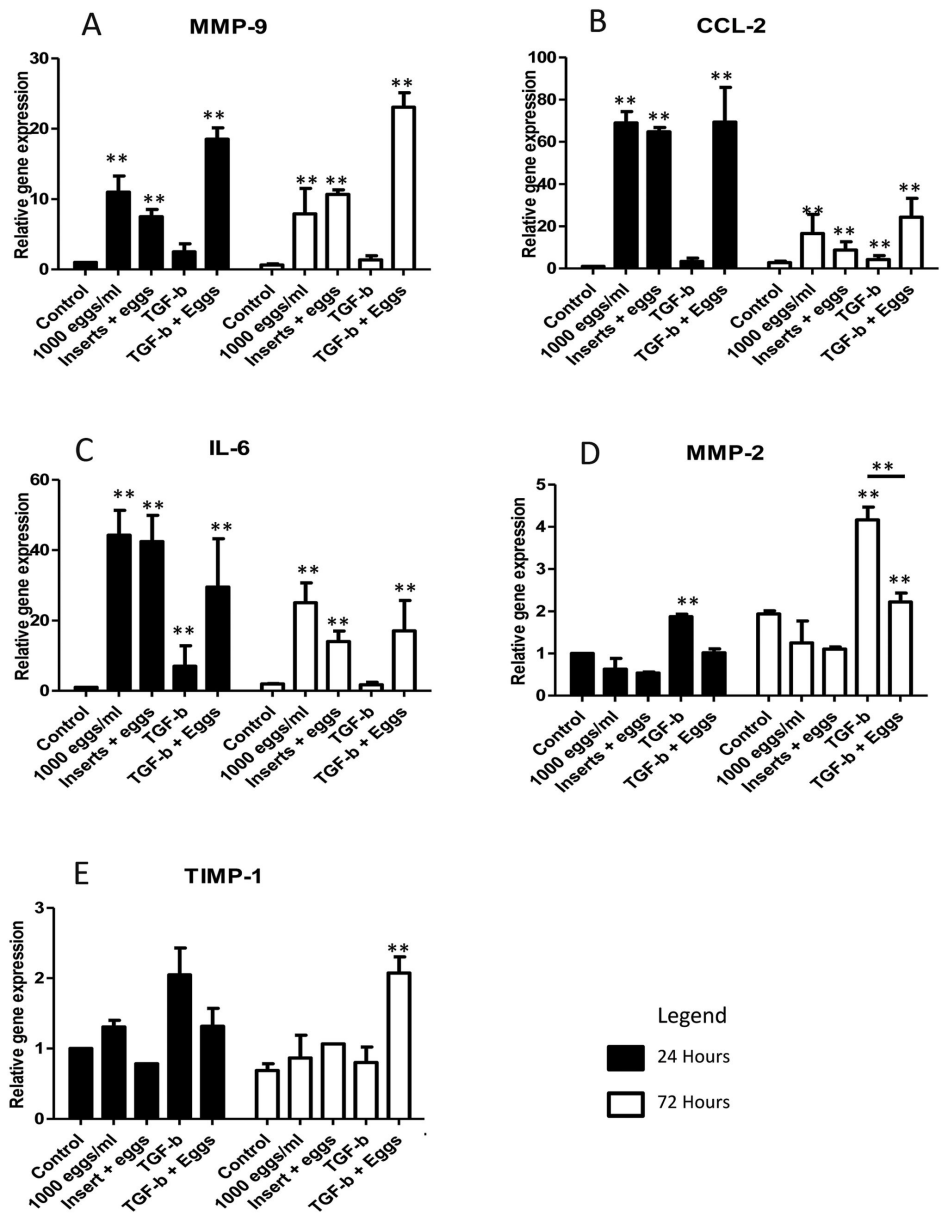
Effect of *S*. *japonicum* eggs on the expression of genes associated with matrix re-organisation and inflammation in LX-2 cells. Expression of genes associated with matrix re-organisation and inflammation; A. MMP-9, B. CCL2, C. IL-6, D. MMP-2 and E. TIMP-1, were measured for gene expression relative to the housekeeping genes, YWHAZ and ATP5b. Eggs of *S. japonicum* significantly promoted the expression of genes associated with granuloma regulation (MMP-9 (A), CCL2 (B) and IL-6 (C)) compared with untreated cells. TGF-β treatment of cells resulted in significantly increased expression of MMP-2 (D) and MMP-9 (A), at both 24 and 72h, and TIMP-1 (E) at 24h. (n=3, SEM).

Of genes associated with ECM maintenance, MMP-2 expression remained similar to control levels and TIMP-1 was marginally, although not significantly, increased in egg-treated cells. However, there was a significant increase in expression of MMP-9 in cells in direct contact with eggs (10-fold) as well as cells incubated with eggs separated by inserts (7-fold) (Figure 2 A, D and E). MMP-2 expression was increased by TGF-β stimulation at 24 and 72 h (2- and 4-fold) but co-treatment with TGF-β and eggs blocked this effect (Figure 2D). TGF-β resulted in an increase in expression of MMP-9 compared with controls, but this was dwarfed when compared with the response of eggs alone or egg with TGF-β co-treatment (Figure 2A). The regulation of these genes by eggs suggests their ability to stimulate HSCs to remodel the ECM which may contribute to granuloma formation.

Notably, genes associated with inflammation (CCL2 and IL-6) were substantially up-regulated in egg-treated cells and eggs + TGF-β co-treatment at 24 h (CCL2, ~70-fold; IL-6, ~40-fold) and with increased expression also seen after 72h (~10-20-fold) (Figure 2B and C). This would indicate a role for HSCs in inflammatory cell recruitment in granuloma formation with CCL2 known to cause chemotaxis of both monocytes and HSCs, and IL-6 involved in immune response regulation [23–25].

Taken together, these results indicate that HSC in the immediate region around *S* *. japonicum* eggs are involved in matrix re-modelling, control of cellular influx and immune regulation, but their fibrogenic phenotype is suppressed. This novel phenotype indicates a potential mechanism by which HSCs may play a role in regulating the development of granuloma pathology.

### *S* *. japonicum* eggs induce a more quiescent HSC morphology

**Figure 3:**
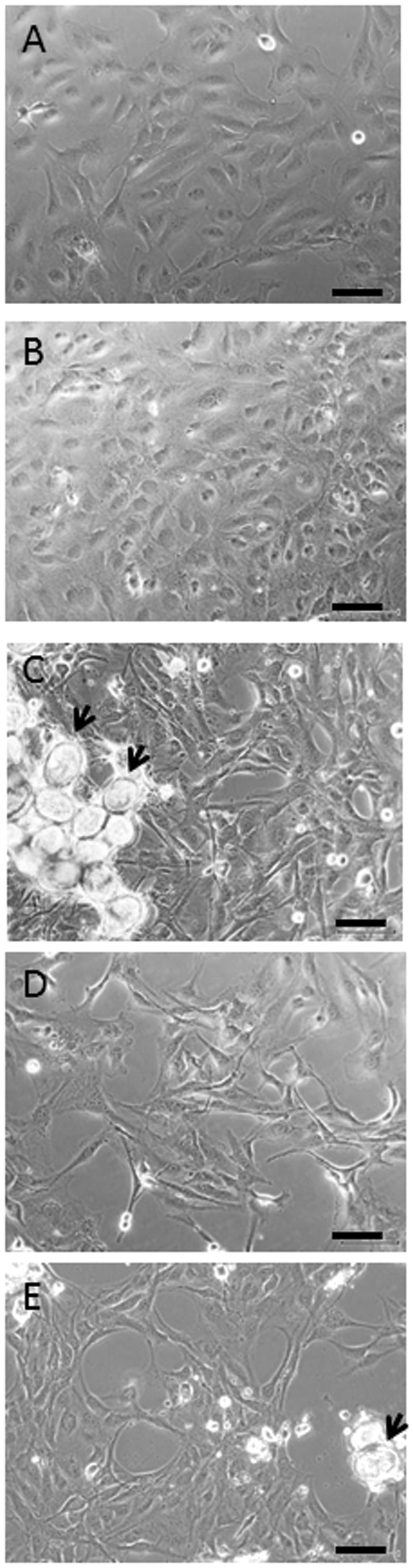
Phase contrast imaging of LX-2 cells. LX-2 cells grown for 72hs (A) under normal cell culture conditions or treated with (B) TGF-β alone display an activated myofibroblast phenotype with a large, flat appearance. Cells, in (C) direct contact with *S. japonicum* eggs (arrows), (D) separated from the eggs by inserts or (E) co-treated with eggs + TGF-β (E) leads to the cells becoming smaller, slender and more elongated with some exhibiting cellular processes by 72h. Scale bars represent 50 µm.

**Figure 4:**
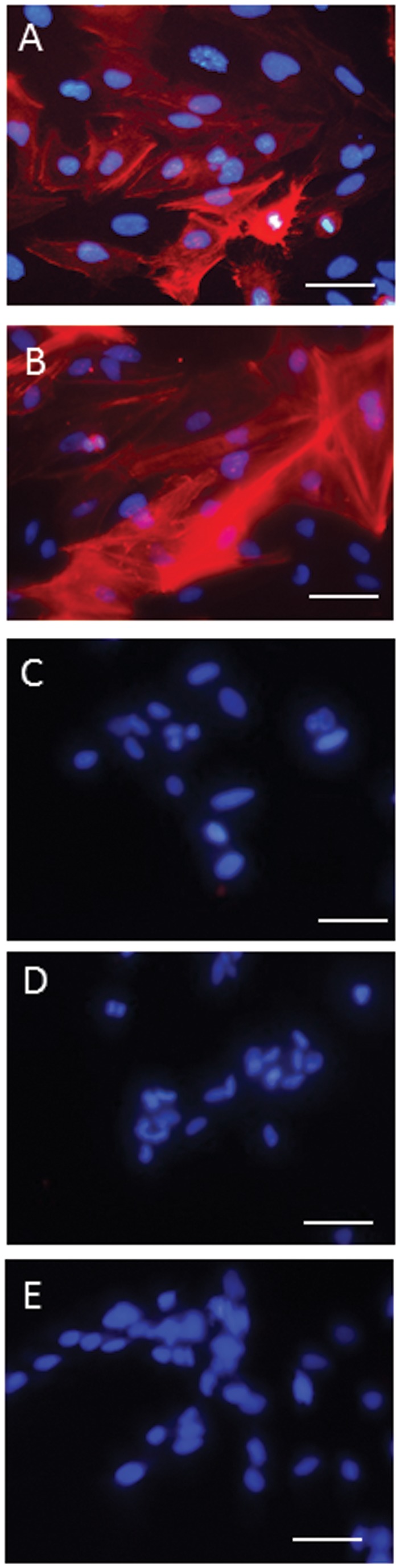
Immunocytochemistry of αSMA in LX-2 cells. LX-2 cells grown for 72h under (A) normal cell culture conditions or treated with (B) TGF-β alone display an activated myofibroblast phenotype with cells staining positively for αSMA stress fibres (red). Cells, in (C) direct contact with *S. japonicum* eggs, (D) separated from the eggs by inserts, or (E) co-treated with eggs + TGF-β leads to the cells losing positive staining for αSMA. Cell nuclei are stained blue by DAPI. Scale bars are 50µm.

LX-2 cells exhibited an activated HSC phenotype under normal cell culture conditions on tissue culture plastic, characterised by the cells having a large rounded appearance (Figure 3A) with a prominent stress fibre staining pattern observed when stained for αSMA (Figure 4A). However, cells treated with eggs either in direct contact or with eggs in inserts, had a more quiescent phenotype with a more slender appearance (Figure 3B and C) and did not exhibit stress fibre staining patterns with αSMA staining (Figure 4B and C); indeed, little staining for αSMA was observed in egg-treated cells.

### *S* *. japonicum* eggs fail to induce lipid droplet retention

**Figure 5:**
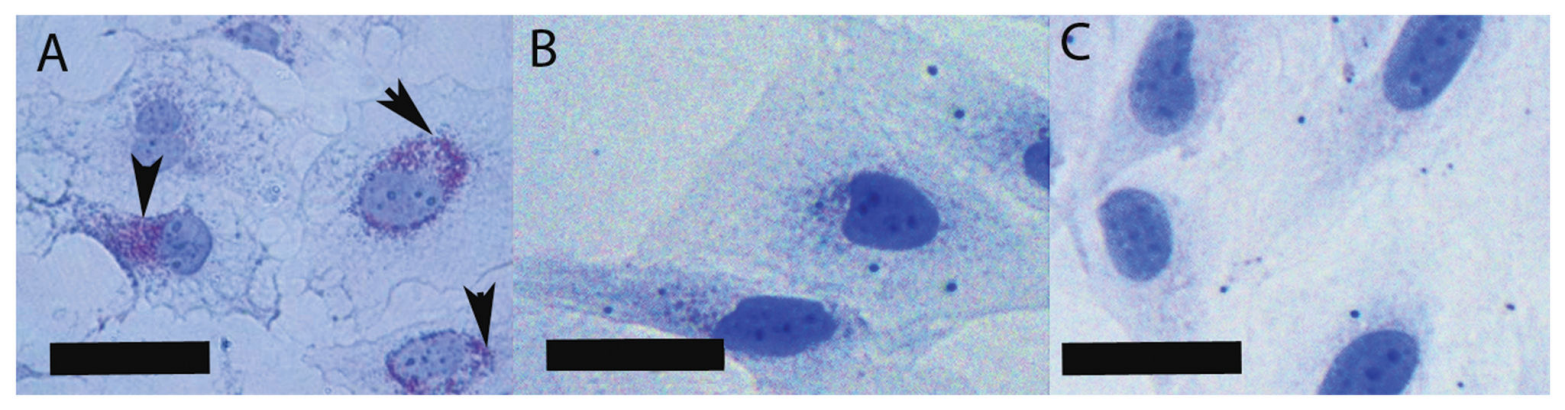
Lipid droplet staining of LX-2 cells. LX-2 cells grown for 72h were stained for lipid droplets by Oil Red O staining. A (A) positive control of LX-2 cells treated with MDI to induce lipid droplet storage, as described in previous experiments [13,15], was stained and observed to stain for lipid droplets (arrows). No evidence of lipid droplet staining was observed in the LX-2 cells cultured in (B) normal cell culture conditions or it the (C) presence of *S. japonicum* eggs. Scale bars are 50µm.

As *S* *. japonicum* egg-treated LX-2 cells exhibit a more quiescent morphology, and previous studies have shown that HSCs treated with *S. mansoni* eggs restores the lipid droplet storing potential of HSCs, we stained for lipid droplets using Oil Red O. However, only a marginal increase in lipid droplet retention was observed between *S* *. japonicum* egg-treated for 24 or 72h (Figure 5B and C) and control cells (Figure 5A), suggesting that although a more quiescent phenotype was observed in the cells, complete quiescence was not induced.

### *S* *. japonicum* eggs reverse TGF-β-mediated HSC myofibroblastic morphology and phenotype

Treatment of the LX-2 cells with TGF-β results in an activated phenotype. Cells were large and rounded (Figure 3D) and were observed to have a stress fibre staining pattern for αSMA (Figure 4D). Co-treatment with TGF-β + eggs resulted in a reversion of this phenotype with the cells demonstrating a more slender phenotype (Figure 3E) and little staining for αSMA (Figure 4E), a feature associated with quiescent cells.

## Discussion

This study demonstrates for the first time a specific granuloma regulatory phenotype in HSCs caused directly by interaction with soluble mediators produced by eggs of the parasite *S* *. japonicum* . This is associated with the HSCs having their fibrogenic potential switched off while expressing high levels of MMP-9, CCL2 and IL-6 which would enable them to remodel ECM, attract monocytes and additional HSCs proximal to the egg, as well as regulating the immune response around it.

Myofibroblasts derived from HSCs are one of the most important cell types associated with fibrogenesis in the liver [12]. HSCs have been demonstrated to play a role in fibrogenesis in schistosomiasis caused by both *S* . japonicum [9] and *S. mansoni* [10]. However, a recent study showed that *S. mansoni* eggs were able to reverse the activated HSC myofibroblastic phenotype back into quiescent lipid-storing cells [15]. We have now demonstrated this may also be the case in *S* *. japonicum* infection with LX-2 cells expressing significantly reduced levels of αSMA and Col1A1 when treated with parasite eggs. However, unlike the situation with *S. mansoni*–egg treated cells, restoration of lipid droplet retention and increased expression of the quiescence marker PPARγ were not observed. Thus, in contrast to the pathology associated with *S. mansoni*, the reduced ability of *S* *. japonicum* eggs to induce a quiescent phenotype may, at least in part, explain why schistosomiasis japonica is a much more pathogenic disease. Differences between the two species are summarised in Table 1. Notably, this phenomenon was observed in cells both in direct contact with *S* *. japonicum* eggs and in cells co-cultured away from the eggs in inserts, suggesting this effect is mediated by a secreted product of the eggs. This result may explain why fibrosis within the granuloma is only observed around its periphery and not around the immediate vicinity of the eggs with eggs blocking fibrogenesis in HSCs immediately around them while HSCs further away, being exposed to reduced levels of egg antigens, become activated as a result of the host immune response, likely due to high levels of IL-13. Fibrosis severity correlates with IL-13 levels and is reduced by expression of the IL-13 decoy receptor, IL-13Rα2, or by neutralising antibodies for IL-13 in a murine model of *S. mansoni* [26]. The cytokine is produced in high levels by TH2 type CD4+ T cells and alternatively activated macrophages [27]. There is also evidence that due to the “sticky” nature of schistosome egg secretions, that egg antigens may be sequestered in the vicinity of the egg [28].

**Table 1:** Summary of the different effects resulting from the treatment of LX-2 cells with *S. mansoni* or *S. japonicum* eggs.

	**Activated LX-2 Cells**	**Quiescent LX-2 Cells**	**LX-2 cells treated with *S. mansoni* eggs**	**LX-2 cells treated with *S. japonicum* eggs**
**Stress fibre staining**	+**ve**	**-ve**	**-ve**	**-ve**
**Lipid droplet staining**	**-ve**	+**ve**	+**ve**	**-ve**
**αSMA mRNA expression**	**High**	**low**	**low**	**low**
**Col1A1 mRNA expression**	**High**	**low**	**low**	**low**
**PPARγ mRNA expression**	**low**	**high**	**high**	**low**

The table summarises the differences between LX-2 cells in their activated and quiescent phenotypes and their features after treatment with eggs of *S. mansoni* (data derived from Anthony et al. (15)) or *S* *. japonicum* . *S. mansoni* eggs induce a fully quiescent phenotype but whereas eggs of *S* *. japonicum* inhibited the fibrogenic phenotype of the LX-2 cells, they failed to induce the adipogenic part of the quiescent phenotype.

This study has expanded our previous findings [29] in that we have investigated the expression of *S* *. japonicum* genes associated with matrix re-organisation and inflammation alongside the transdifferentiation-associated genes. *S* *. japonicum* eggs induce the ability of HSC to re-organise the surrounding matrix. The eggs induced high levels of MMP-9 expression in the absence of increased expression of MMP-2 or TIMP-1. This would suggest that MMP-9 may be able to digest the basement membrane in the vicinity of the eggs unimpeded by its inhibitors. This supports the demonstration that myofibroblasts isolated from granuloma tissue express MMP-9 but not MMP-2, which was found to be produced by other cell types [30]. In the liver, where schistosome eggs are trapped, MMP-9 expression may facilitate the influx of inflammatory cells. MMP-9 expression is essential for recruitment of neutrophils and T-cells in a model of post-ischemic liver disease [31]. MMP-9 was demonstrated to be involved in early recruitment cascades of neutrophils with inhibition of MMP-9 resulting in reduced migration of these cells [31]. This was also shown by others where MMP-9 deficiency resulted in reduced leukocyte traffic, as well as disrupted neutrophil migration across fibronectin in transwell filters [32]. This supports our observations *in vivo* with neutrophils located around the egg in the granuloma of BALB/c mice infected with *S* *. japonicum* in a previous study [29]. MMP-9 has, additionally, been demonstrated to have a role in macrophage migration. Plasminogen is known to activate MMP-9 expression and its knockout in mice results in reduced MMP-9 expression along with reduced macrophage migration in a murine model of inflammation [33,34] ; by administering MMP-9 to mice macrophage migration was restored [34].

CCL2 and IL-6 expression were extensively up regulated by *S* *. japonicum* egg treatment of LX-2 cells, both in direct contact with eggs and when separated using inserts, suggesting the cells have an ability to regulate inflammatory cell infiltration and thus the local immune response proximal to the egg. Fibroblasts isolated from schistosome granuloma tissue have been demonstrated to express CCL2 [33]. Although CCL2 seems to have a relatively low expression in *in vivo* models of disease, it has been demonstrated to play an important role in early granuloma regulation [35]. This is supported in other non-granulatomous liver models; for example, in rodent models of cholestatic liver disease increased expression of CCL2 is observed three days after bile duct ligation, with CCL2 expression increasing prior to both αSMA and Col1A1 [25]. CCL2 is a chemoattract for monocytes, but is also a potent chemokine for HSCs [25]. A key event in early fibrogenesis in cholestatic liver disease is up regulation of CCL2 which results in HSC recruitment [25]. By inhibiting CCL2 production in a bile duct ligated model of liver fibrosis in rats, HSC chemotaxis was markedly reduced [25]. Schistosome egg-stimulated production of CCL2 early in granuloma formation may be involved in monocyte recruitment and recruitment of HSCs into the granuloma area. An influx of activated HSC’s into the granulomas periphery would allow for the collagen accumulation in this region.

IL-6 is important in granuloma regulation and is strongly expressed at the onset of egg laying in the baboon model of *S. mansoni* infection [36]. IL-6 has been demonstrated to have an anti-inflammatory role in schistosomiasis, down-regulating TH-1 responses by inducing the expression of IL-10 which in turn down-regulates expression of INF-γ [23]. This down-regulation of the TH-1 response is one mechanism whereby schistosome eggs are thought to manipulate the host response in order to establish a TH-2 response. Increased expression of IL-6 has been observed in splenocytes stimulated with SEA from *S* *. japonicum* as part of a TH17 response in these cells [37].

A striking feature of this interaction is the ability of schistosome eggs to switch off the response of the HSCs to TGF-β, the most potent fibrogenic activator of these cells [38]. Treatment of LX-2 cells with TGF-β resulted in increased expression of the fibrosis-associated genes, αSMA, Col1A1, CTGF, MMP-2, CCL-2 and IL-6. The cells also demonstrated a more activated phenotype, being observed to stain heavily for αSMA stress fibres. However, upon co-treatment with both TGF-β and eggs, the expression of αSMA and Col1a1 was inhibited and was similar to the expression levels observed in the presence of eggs alone. Additionally, LX-2 cells exhibited a more quiescent phenotype as observed morphologically by phase contrast microscopy as well as the lack of αSMA stress fibres. Expression of MMP-2 was not increased by co-treatment with TGF-β and eggs and was lower in comparison with TGF-β treatment alone. Notably, the levels of CTGF remained at a high level of expression indicating that the CTGF response to TGF-β was not lost and that inhibition of the pro-fibrogenic response may occur downstream of CTGF expression. This may also have some effect on the granuloma formation process as CTGF expression is increased in the granuloma and liver of schistosome-infected mice [29]. It may be these cells can produce CTGF in response to latent TGF-β in the ECM which acts in the periphery of the granuloma resulting in the activation of HSC leading to fibrosis in this area without an increase in TGF-β levels. CTGF can be stimulated in rat HSCs by IL-13 through activin receptor-like kinase/Smad signalling via the Erk-MAPK pathway [8]. The ability of the eggs to switch off the response to TGF-β in LX-2 cells supports the fact that fibrosis in schistosomiasis is considered to be independent of TGF-β and driven by IL-13 expression [39].

Precisely how schistosome eggs interact with HSCs remains unknown. However some evidence of altered TLR-4 signalling may be involved as expression of genes linked to NF-κB activation, CCL2, MMP-9 and IL-6, are greatly increased in this model. It has been demonstrated that the glycan lacto-N-fucopentose III (LNFPIII), isolated from secreted egg antigen preparations of *S. mansoni*, drives a dendritic cell type 2 phenotype which is dependent on TLR-4 signalling [40]. It has additionally been demonstrated that this activation of NF-κB by LNFPIII is different to that stimulated by LPS as it was only produced in an initial response and not a prolonged response [41], although the precise mechanism of action is as yet unknown. Previous studies have demonstrated that LPS-induced TLR-4 stimulation in HSCs renders LX-2 cells hypersensitive to TGF-β [42] mediated by LPS down regulation of the TGF-β pseudo-receptor Bambi, which sensitises the cells to TGF-β stimulation allowing for unrestricted HSC activation [42]. However, in the current study, the profibrogenic response of the cells to TGF-β was lost suggesting that if TLR-4 signalling occurs, an alternative pathway of NF-κB activation may be involved.

**Figure 6:**
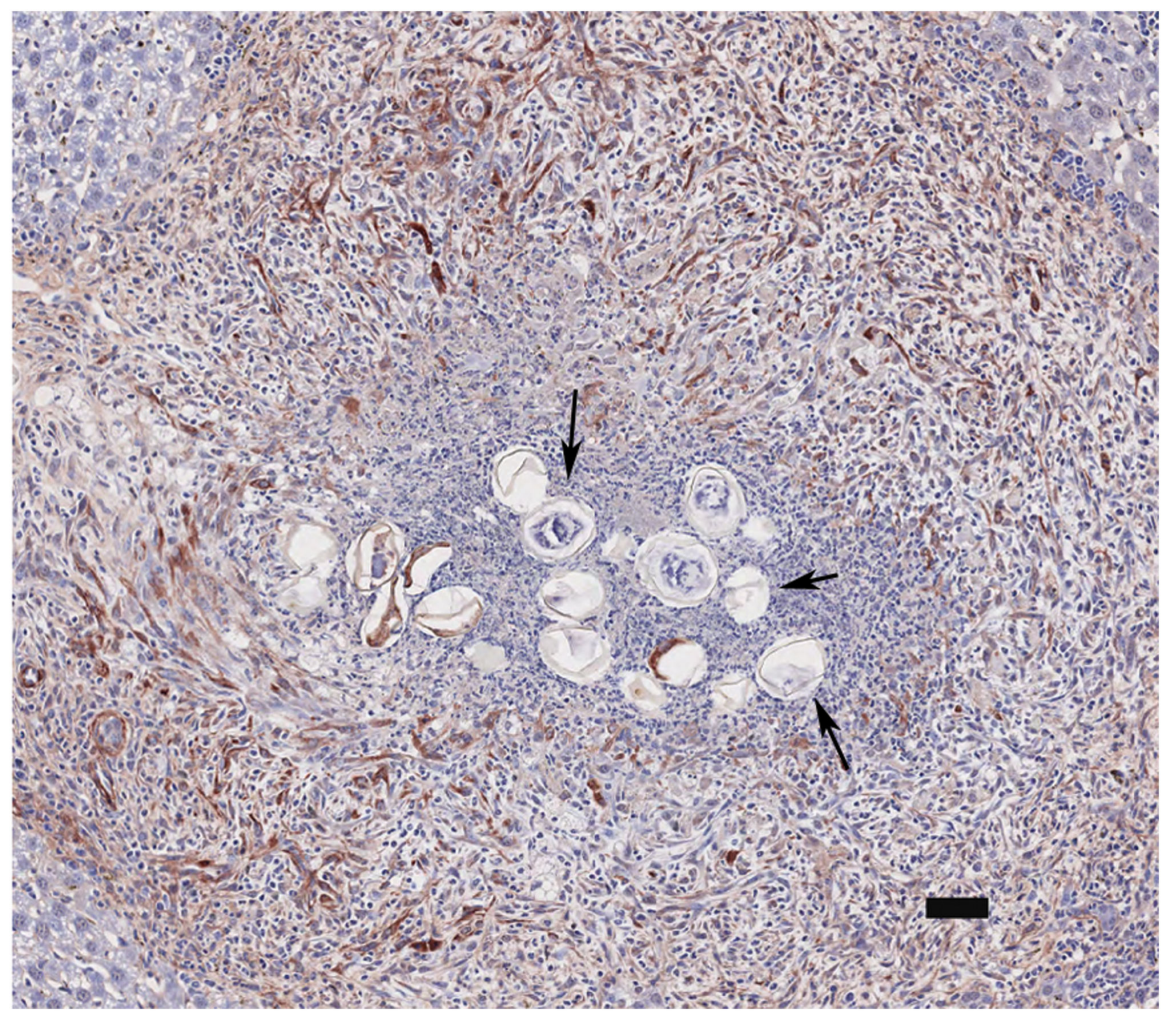
αSMA staining of murine granuloma induced by *S*. *japonicum*. This image displays a granuloma stained for a marker of activated HSC, alpha-smooth muscle actin (αSMA), in a murine model of schistosomiasis japonica taken from our image library. Positive staining for αSMA (brown) can clearly be observed around the granuloma periphery but not in the immediate vicinity of the eggs (arrows). Our results indicate that HSCs in the immediate vicinity of eggs would have reduced αSMA staining and reduced collagen production due to the antifibrotic influence of the eggs, while HSC in the granuloma periphery would be activated and observed to express αSMA due to the host profibrogenic response in the absence or reduced concentration of egg antigen. Scale Bar is 50µm.

In conclusion we hypothesise that the eggs of *S* *. japonicum* down-regulate the process of fibrogenesis in HSC while at the same time promoting early inflammation and processes that regulate the formation of granulomas around the eggs. Collagen is localised in the periphery of the granuloma and not around the immediate vicinity of the egg, where secreted antigens/proteins would inhibit or reverse the activation of HSCs. We propose that cell recruitment into the granuloma area by monocytes and HSCs occurs due to CCL2, as facilitated by matrix re-organisation driven by MMP-9 production. Activated HSC’s would be observed at the periphery of the granuloma (Figure 6). Immune regulation may occur with IL-6 driving the production of IL-10 which in turn would result in the down-regulation of the TH-1 response and facilitate the establishment of a TH-2 response. The exact mechanisms stimulating this phenotype are unknown and it is important to investigate the nature of this interaction and our hypothesis further as this may provide additional pointers regarding the key events in early granuloma formation. This will deepen our understanding on the pathology associated with the disease of schistosomiasis which may aid in the development of novel anti-pathology drugs or vaccines. Additionally, furthering our understanding of the process whereby fibrogenesis in HSCs is switched off may reveal future drug targets for anti-fibrotic effects in other liver disease characterised by fibrosis.
